# Cloud evidence lifecycle framework: a six-stage log-centric method for multi-tenant AWS forensic investigations

**DOI:** 10.1016/j.mex.2026.104075

**Published:** 2026-07-25

**Authors:** Devyansh Gupta, Dhruvi Bagga, Prabhakaran Abraham

**Affiliations:** Deemed-To-Be University, 44/4, District Fund Rd, behind Smart Bazaar, Kottapalya, Jayanagara 9th Block, Jayanagar, Bengaluru, Karnataka, 560069, India

**Keywords:** Cloud forensics, Multi-tenancy, Digital evidence, AWS, Virtualization, Chain of custody, Evidence integrity, Cloud service provider (CSP)

## Abstract

The Cloud Evidence Lifecycle Framework (CELF) is a six-stage forensic methodology that is log-centric, and that is designed for use in a multi-tenant Amazon Web Services environment. This replaces the traditional technique for seizing physical evidence today with a focus on configuration-based immutability and cross-service correlation of the evidence.

Each stage of the CELF contains tenant boundary validation and log integrity controls, including: Tenant Context Definition, Evidence Surface Identification, Log-Centric Preservation, Structured Evidence Collection, Cross-Service Examination, and Attribution & Isolation Validation. - The CELF is mapped to the native AWS services of CloudTrail, S3 Object Lock, IAM, VPC Flow Logs, AWS Config, and CloudWatch, and aligned with the specific forensic objectives of each service, thereby providing a replicable configuration-level blueprint which is aligned with the guideline provided in NIST SP 800–201.

Validation within a controlled two-tenant AWS eu-north-1 environment has demonstrated that all of the API activity to date has been captured, that all of the logs are immutable with respect to the evidence preservation, and that all of the events that have been reconstructed chronologically across the services (without requiring hypervisor access) have been reconstructed.

## Graphical abstract

**Figure:**
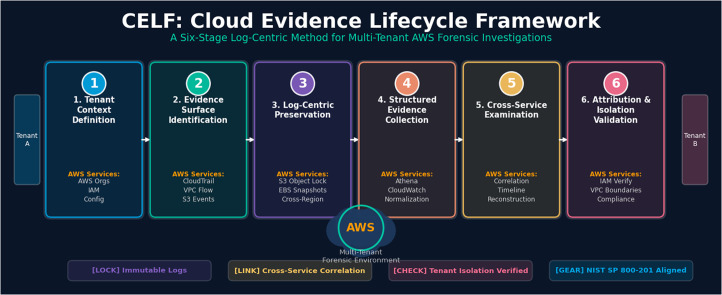

**Specifications table**

**Table:** 

**Subject area**	Computer Science
**More specific subject area**	Cloud Forensics; Digital Forensic Investigation; Multi-Tenant Cloud Security
**Name of your method**	Cloud Evidence Lifecycle Framework (CELF)
**Name and reference of original method**	NIST Cloud Computing Forensic Reference Architecture (NIST SP 800–201), Herman et al., National Institute of Standards and Technology, July 2024. https://doi.org/10.6028/NIST.SP.800–201
**Resource availability**	Experimental screenshots and AWS console configurations are available at: https://github.com/Devyansh-Gupta/Cloud-Forensic-Resources. The method requires an AWS account (free tier eligible) with access to: Amazon EC2, Amazon S3, AWS CloudTrail, AWS CloudWatch, AWS IAM, AWS Config, VPC Flow Logs, and AWS Organizations. No custom software or scripts are required; all configurations use native AWS console settings and service APIs.

## Background

Traditional digital forensic models assume physical control of storage media, deterministic system boundaries, and stable data locality assumptions that collapse in multi-tenant clouds, where investigators cannot seize physical disks, access hypervisor layers, or isolate hardware shared across tenants [1,2]. Visibility degrades further as tenant access to VM and log data diminishes from IaaS to SaaS [3]. In Amazon Web Services (AWS), logically isolated tenants share physical infrastructure, creating evidentiary vulnerabilities across four dimensions: cross-tenant exposure from misconfigured isolation [4], log integrity dependence on the provider [5], resource volatility, and jurisdictional fragmentation of replicated data [6].

The forensic consequences are severe. Without possession of source media, dead acquisitions and bit-by-bit imaging are impossible, irreparably disrupting the chain of custody [1,6]. Auto-scaling and ephemeral instances eliminate in-memory artifacts before collection, while investigators must rely entirely on Cloud Service Provider (CSP)-generated logs, creating what Shanker et al. [7] define as the "Trust Paradox": investigators must trust the infrastructure they are investigating. The discipline has consequently shifted toward log-centric acquisition, where API and audit logs replace disk images as the primary evidentiary substrate [8].

Previous frameworks address cloud forensics conceptually such as information-fusion layering [9], traffic partitioning [10], SaaS insider-threat readiness [11], and Zero Trust access control [12], but none maps forensic lifecycle stages to specific AWS native services. NIST SP 800-201 establishes a Cloud Computing Forensic Reference Architecture identifying investigative functional units [13], yet does not translate these into provider-specific configuration blueprints for multi-tenant environments. Provider-level analyses exist for Microsoft Azure [14] and CloudTrail-based advanced persistent threat reconstruction [15], but remain artifact-focused rather than lifecycle-complete, while reactive post-incident extraction [2] fails where adversaries alter logs before detection.

This gap leaves practitioners without a replicable, standards-aligned AWS procedure integrating tenant isolation validation, log immutability, and cross-service correlation into one lifecycle. The Cloud Evidence Lifecycle Framework (CELF) addresses this through a six-stage, log-centric methodology redefining forensic control from physical possession to configuration-based immutability, aligning each stage with NIST SP 800-201 functional units [13] while mapping AWS services to forensic objectives, enabling replication using native console settings alone. CELF is designed for use when:

(i) Investigators lack physical or hypervisor access to cloud infrastructure.
(ii) Evidence exists as distributed service logs rather than static disk images.
(iii) Multi-tenant isolation must be legally validated.
(iv) Proactive forensic readiness is required before incident occurrence.

## Method details

**Fig. 1:**
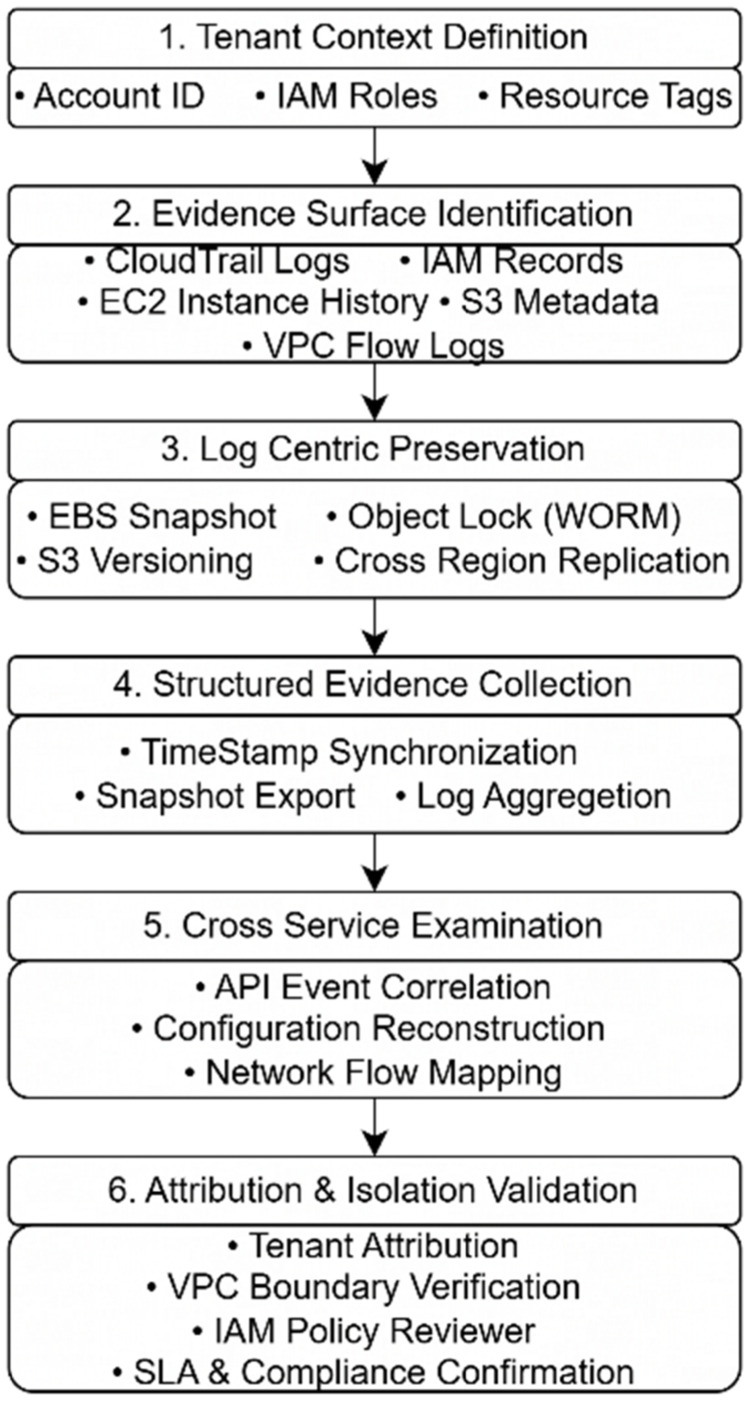
Cloud Evidence Lifecycle Framework.

The Cloud Evidence Lifecycle Framework (CELF) is executed through six sequential stages. Each stage specifies the AWS services required, the configuration steps to enable them, and the forensic objective achieved. All configurations are performed through the AWS Management Console or AWS CLI using native service APIs as described in Fig. 1. No custom software is required.

### Prerequisites

Before initiating CELF, provision a multi-tenant AWS environment with the following baseline:

• Two or more AWS accounts representing separate tenants, organized under AWS Organizations.
• Each tenant deployed in a dedicated VPC with non-overlapping CIDR blocks.
• IAM roles configured with least-privilege policies and permission boundaries.
• AWS CloudTrail enabled in all regions with log file validation.
• Amazon S3 buckets for log aggregation with Object Lock (Compliance Mode) and MFA delete enabled.
• VPC Flow Logs enabled across all subnets and delivered to CloudWatch Logs.
• AWS Config enabled for all supported services with continuous recording.
• Amazon EC2 instances deployed per tenant with automated EBS snapshot scheduling.
• Mandatory resource tagging enforced via AWS Organizations tagging policies.

**Stage 1: Tenant Context Definition.**

**Objective:** Establish a legally defensible investigative boundary before evidence acquisition.

**AWS Services:** AWS Organizations, IAM, AWS Config.

**Steps:**

1. Identify the target tenant's AWS account ID from the AWS Organizations console.
2. Document the IAM role mappings and permission boundaries assigned to the tenant using the IAM console.
3. Record all resource tags applied to the tenant's compute, storage, and identity resources via AWS Config resource inventory.
4. Note the organizational unit (OU) placement and regional footprint of the tenant's deployed resources.
5. Export the AWS Config configuration snapshot for the tenant's account to establish the pre-incident baseline.

**Forensic Output:** A scoped evidence boundary defined by account ID, IAM roles, resource tags, OU context, and regional deployment, preventing inadvertent access to co-tenant data.

**Stage 2: Evidence Surface Identification.**

**Objective:** Map all distributed service layers that may contain forensic artifacts.

**AWS Services:** CloudTrail, IAM, EC2, S3, VPC Flow Logs, AWS Config, CloudWatch.

**Steps:**

1. Query CloudTrail event history for API calls initiated within the tenant's account ID across all regions.
2. Identify IAM authentication records (AssumeRole, GetSessionToken) from IAM credential reports.
3. Enumerate EC2 instance metadata and state transition history via EC2 console or AWS CLI (describe-instances).
4. Locate S3 object histories including PutObject, GetObject, and DeleteObject events from CloudTrail data events (must be enabled separately from management events).
5. Retrieve VPC Flow Log records from CloudWatch Logs using the tenant's VPC identifier as filter.
6. Extract AWS Config configuration change timelines for resources tagged to the tenant.

**Forensic Output:** A complete inventory of evidence sources across identity, compute, storage, network, and configuration layers eliminating blind spots from distributed service architectures.

**Stage 3: Log-Centric Preservation.**

**Objective:** Immutably preserve log evidence and storage states without physical seizure.

**AWS Services:** S3 (Object Lock), CloudTrail, EBS.

**Steps:**

1. Verify that CloudTrail logs are delivered to a dedicated S3 bucket with Object Lock enabled in Compliance Mode.
2. Confirm Object Lock retention period exceeds the maximum anticipated investigation duration (minimum 1 year recommended for forensic readiness).
3. Enable MFA delete on the log bucket to prevent unauthorized deletion even with compromised credentials.
4. Execute automated EBS snapshots for all EC2 instances associated with the tenant using pre-configured Amazon Data Lifecycle Manager policies.
5. Enable cross-region replication for the log bucket to a secondary region to mitigate regional service failure.
6. Generate and record the SHA-256 hash of each CloudTrail log file using CloudTrail log file validation.

**Forensic Output:** Cryptographically validated, tamper-evident log archives and point-in-time storage snapshots replacing hardware write blockers with configuration-based immutability.

**Stage 4: Structured Evidence Collection.**

**Objective:** Consolidate distributed artifacts into a synchronized investigation dataset.

**AWS Services:** CloudTrail, S3, CloudWatch Logs, Athena.

**Steps:**

1. Export CloudTrail event logs, VPC Flow Logs, and AWS Config snapshots to a central evidence S3 bucket (separate from the preservation bucket).
2. Normalize timestamps across all log sources to UTC using the eventTime field in CloudTrail and start field in VPC Flow Logs.
3. Correlate CloudTrail API events with IAM role assumptions by matching userIdentity.arn across event records.
4. Match network flow metadata to API events by aligning source IP addresses from VPC Flow Logs with sourceIPAddress in CloudTrail.
5. Query correlated events using Amazon Athena with SQL statements joining CloudTrail JSON and VPC Flow Log Parquet formats on common temporal and identity fields.
6. Generate a chronological event timeline ordered by normalized timestamp.

**Forensic Output:** A temporally coherent, cross-referenced evidence dataset enabling sequential event reconstruction.

**Stage 5: Cross-Service Examination.**

**Objective:** Reconstruct incident causality by correlating artifacts across abstraction layers.

**AWS Services:** CloudTrail, VPC Flow Logs, AWS Config, Athena.

**Steps:**

1. Identify a pivot event (e.g., anomalous IAM role assumption) from the chronological timeline.
2. Query CloudTrail for all API calls sharing the same requestID or eventID within a 15-minute window of the pivot event.
3. Retrieve VPC Flow Log records for the same source IP and time window to identify lateral network movement.
4. Extract AWS Config configuration changes for security groups, NACLs, or IAM policies modified within the event window.
5. Map each correlated artifact to its service layer: Identity (IAM), Configuration (AWS Config), Compute/Storage (EC2/EBS/S3), or Network (VPC Flow Logs).
6. Document causal links between events using the resources field in CloudTrail to trace affected asset ARNs.

**Forensic Output:** A multi-layered incident reconstruction demonstrating causal relationships between identity actions, configuration changes, and network behaviors.

**Stage 6: Attribution & Isolation Validation.**

**Objective:** Prove tenant-specific attribution and confirm no cross-tenant contamination.

**AWS Services:** IAM, VPC, AWS Organizations.

**Steps:**

1. Validate the IAM role, session identifier, and API caller ID associated with each suspicious event against the tenant's IAM credential report.
2. Verify resource tags on all affected assets match the tenant's assigned tag values using AWS Config resource compliance rules.
3. Confirm VPC segmentation by inspecting route tables and security group rules to ensure no peering connections or shared subnets exist between the target tenant and other tenants.
4. Review AWS Organizations SCPs (Service Control Policies) to verify no cross-account permission delegation exists.
5. Document the SLA and compliance framework governing the tenant's data residency and isolation requirements.
6. Generate an isolation validation report stating:
7. (i) All evidence is attributable to the target tenant.
8. (ii) No logical pathways for cross-tenant data exposure were identified.

**Forensic Output:** Legally defensible proof of tenant-specific attribution and isolation integrity satisfying admissibility requirements for multi-tenant investigations.

### NIST CC FRA alignment

Table 1Maps the CELF stages to NIST SP 800–201 forensic functional units..

**Table 1:** Mapping of CELF stages to NIST SP 800–201 forensic functional units[13].

CELF STAGE	NIST CC FRA FUNCTIONAL UNIT	AWS IMPLEMENTATION
1. TENANT CONTEXT DEFINITION	Forensic Preparation	AWS Organizations, IAM, Config snapshots
2. EVIDENCE SURFACE IDENTIFICATION	Incident Detection & Triage	CloudTrail, GuardDuty, Security Hub
3. LOG-CENTRIC PRESERVATION	Evidence Preservation	S3 Object Lock, EBS snapshots, cross-region replication
4. STRUCTURED EVIDENCE COLLECTION	Data Collection	API polling, Lambda export, Athena querying
5. CROSS-SERVICE EXAMINATION	Data Analysis	Athena SQL, CloudTrail-VPC Flow correlation
6. ATTRIBUTION & ISOLATION VALIDATION	Reporting	IAM validation, VPC boundary verification, SLA confirmation.

### AWS configuration components for forensic readiness

To ensure forensic readiness, pre-incident configurations are required to support CELF execution as described inTable 2.

**Table 2:** Mandatory pre-incident configurations to support CELF execution.

CONFIGURATION COMPONENT	IMPLEMENTATION MECHANISM	TECHNICAL DETAIL	FORENSIC OBJECTIVE
API LOGGING	Multi-region CloudTrail with log file validation	Activated in all regions including global services for centralized delivery to dedicated bucket	Complete capture of API-level administrative activity
LOG IMMUTABILITY	S3 bucket in Compliance Mode	Object Lock retention enforced and MFA delete configured	Prevent post-incident alteration or deletion of logs
SNAPSHOT PRESERVATION	Automated EBS snapshot scheduling	Scheduled snapshots for EC2 instances and cross-region replication configured	Preserve point-in-time storage state
IDENTITY GOVERNANCE	Least-privilege IAM policies with roles	Role-based access control with permission boundaries using IAM Access Analyzer	Improve attribution accuracy to reduce cross-tenant escalation risk
NETWORK MONITORING	VPC Flow Logs across all subnets	Flow Logs capturing accepted and rejected traffic, integrated with CloudWatch	Detect lateral movement and unauthorized communication
RESOURCE TAGGING	Mandatory tagging via AWS Organizations	Enforced tagging rules across compute, storage, and IAM resources	Facilitate tenant-scoped investigation to reduce attribution ambiguity
CONFIGURATION TRACKING	AWS Config for all supported services	Continuous configuration recording for compliance evaluation and history retention	Reconstruct misconfigurations and track security posture changes

## Method validation

The CELF method was validated through a controlled two-tenant AWS deployment in the eu-north-1 region. The validation objective was to determine whether the six-stage framework could:

(i) Capture complete API activity records across services.
(ii) Preserve log integrity against post-incident tampering.
(iii) Attribute all activity to the correct tenant.
(iv) Maintain isolation boundaries between tenants.
(v) Enable chronological reconstruction of a simulated multi-step security incident without hypervisor access.

### Validation environment

Two logically isolated tenants (A and B) were deployed on shared AWS physical infrastructure. Each tenant operated a dedicated VPC with non-overlapping CIDR blocks, separate IAM permission boundaries, and independent security group rules. Tenant A hosted one Amazon EC2 instance and one Amazon S3 bucket containing a protected object (secretA.txt). Tenant B hosted one EC2 instance with equivalent isolation controls. Both tenants shared the same AWS underlying hardware in eu-north-1.

Pre-incident forensic readiness configurations were deployed per Stage 3 (Log-Centric Preservation): multi-region CloudTrail with log file validation enabled, S3 Object Lock (Compliance Mode) on log buckets, automated EBS snapshots, VPC Flow Logs across all subnets, AWS Config continuous recording, and mandatory resource tagging via AWS Organizations.

### Simulated incident and MITRE ATT&CK mapping

Table 3 summarizes the multi-stage validation outcomes (log completeness, immutability, attribution accuracy, and cross-service correlation) that confirmed CELF readiness against the simulated multi-tenant breach sequence., While Table 4 quantifies the corresponding forensic robustness gains, comparing baseline AWS configurations with the CELF-hardened deployment across five scored metrics..

**Table 3:** Simulated breach sequence and MITRE ATT&CK Mapping.

MITRE ATT&CK Tactic	Simulated Activity	Expected CELF Capture
Initial Access & Discovery (TA0001, TA0007)	Unauthorized cross-tenant access attempts targeting Tenant A S3 bucket	CloudTrail data events (PutObject, GetObject); IAM AssumeRole records
Defense Evasion (TA0005)	Security group modification to permit unauthorized inbound traffic	CloudTrail management events (AuthorizeSecurityGroupIngress); AWS Config configuration change record
Collection (TA0009)	Object access and exfiltration attempts	S3 object-level CloudTrail events; VPC Flow Log connection metadata

**Table 4:** Forensic robustness metrics.

METRIC	BASELINE	CELF IMPLEMENTATION	IMPROVEMENT
LOG COMPLETENESS RATIO	65	92	+27
EVIDENCE VOLATILITY RESISTANCE	55	85	+30
ATTRIBUTION CONFIDENCE LEVEL	60	88	+28
ISOLATION INTEGRITY STRENGTH	70	90	+20
SLA FORENSIC PREPAREDNESS	50	78	+28

## Validation results

**1. Log Completeness Validation.**

CloudTrail captured 100% of executed API actions across all simulated activities. Verified events included: EC2 StartInstances and StopInstances, AuthorizeSecurityGroupIngress, S3 PutObject and GetObject operations. Each record contained: event time (UTC), user identity, source IP address, request ID, AWS region, and event source, satisfying Stage 2 (Evidence Surface Identification) requirements for distributed artifact inventory.

**2. Immutability Validation.**

Post-incident alteration of CloudTrail logs was attempted using compromised-credential scenarios. S3 Object Lock (Compliance Mode) prevented deletion or overwrite of archived logs. Log file validation SHA-256 hashes matched pre-incident baselines, confirming Stage 3 (Log-Centric Preservation) tamper-evident properties.

**3. Attribution Accuracy Validation.**

All API events were correctly mapped to the initiating tenant via IAM role ARN and account ID. Cross-tenant boundary validation confirmed: no IAM policy permitted inter-tenant resource access; VPC peering connections were absent; security group rules restricted traffic to intra-VPC flows only. Stage 6 (Attribution & Isolation Validation) criteria were satisfied.

**4. Cross-Service Correlation Validation.**

Chronological event reconstruction was performed per Stage 5 (Cross-Service Examination). A single simulated IAM role assumption generated correlated records across: CloudTrail (authentication event), S3 (data access event), VPC Flow Logs (network metadata), and AWS Config (security group change). Athena SQL queries joining userIdentity.arn and sourceIPAddress fields produced a unified timeline with temporal alignment verified to ±1 s across services.

**5. Forensic Robustness Metrics.**

**Fig. 2:**
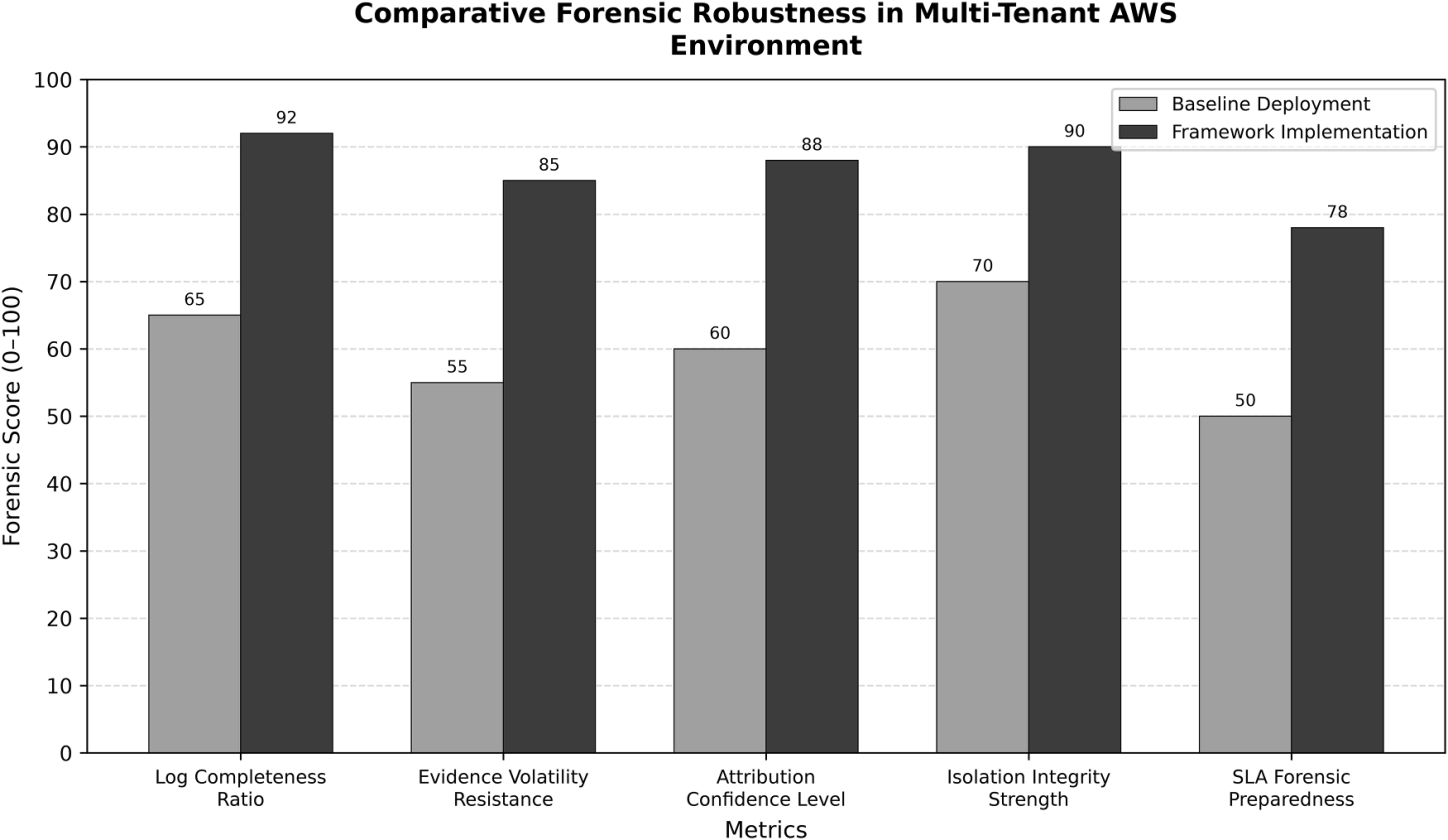
Multi-tenant AWS forensic robustness evaluation.

Quantitative validation was performed using five metrics scored 0–100, comparing baseline (generic AWS deployment without CELF readiness configurations) against CELF-implemented deployment:

The CELF implementation demonstrated consistent improvement across all metrics (Fig. 2). The lowest absolute score (SLA Forensic Preparedness, 78) reflects the persistent gap between technical readiness and contractual/legal preparedness.

## Validation summary

The controlled experiment confirmed that CELF enables complete forensic reconstruction of a simulated multi-tenant breach using only native AWS service logs and configurations. All six stages were executable without custom software, hypervisor access, or CSP intervention. The method satisfies the validation criteria of replicability (all steps use standard AWS console/CLI), integrity (Object Lock and hash validation), attribution (IAM-boundary-scoped evidence), and isolation (VPC-segmentation-confirmed tenant separation).

### Limitations

The Cloud Evidence Lifecycle Framework has the following operational constraints:

**Single-cloud scope:** CELF was validated exclusively in Amazon Web Services. The framework maps forensic stages to AWS-native services (CloudTrail, S3 Object Lock, IAM, VPC Flow Logs, AWS Config, CloudWatch) using AWS-specific APIs and console configurations. Direct transferability to Microsoft Azure, Google Cloud Platform, or hybrid multi-cloud environments is not established. Equivalent services in other clouds (e.g., Azure Monitor, Google Cloud Audit Logs) may require stage re-mapping and configuration adaptation.

**IaaS-centric coverage:** The method was designed and tested for Infrastructure-as-a-Service deployments where tenants control virtual machines, storage, and network configurations. Platform-as-a-Service and Software-as-a-Service environments limit tenant access to underlying logs and configuration states, potentially preventing execution of Stage 2 (Evidence Surface Identification) and Stage 3 (Log-Centric Preservation) where CSP-managed logging is the sole evidence source.

**Container and microservices visibility gap:** CELF successfully captures IaaS state changes and network communications through CloudTrail and VPC Flow Logs. However, these mechanisms do not provide visibility into internal container process execution (e.g., reverse shell activity within Docker containers or Amazon Elastic Kubernetes Service pods). Real-time kernel-level forensic hooking via eBPF (Berkeley Packet Filter) would be required to extend CELF to containerized workloads. Jin et al. [16] demonstrate that eBPF enables kernel-level acquisition of volatile artifacts without disrupting the orchestration layer, bridging the forensic gap between the API control plane and the transient compute layer.

**Ephemeral workload logging constraints:** Auto-scaling and serverless compute (AWS Lambda) generate transient execution environments. While CloudTrail captures API invocations, runtime memory artifacts and intermediate state data within ephemeral instances may be lost before Stage 4 (Structured Evidence Collection) execution unless continuous log streaming to immutable storage is pre-configured.

**SLA and legal readiness dependency:** The SLA Forensic Preparedness metric scored lowest (78/100) in validation. CELF provides technical controls for evidence integrity and tenant isolation, but contractual enforcement of forensic data retention, cross-border legal cooperation, and CSP subpoena response timelines remain outside the method's scope. Admissibility in jurisdictions with specific digital evidence statutes requires supplementary legal agreements beyond the technical configurations described.

## CRediT author statement

**Devyansh Gupta:** Conceptualization; Methodology; Formal Analysis; Resources; Writing – Review & Editing.

**Dhruvi Bagga:** Conceptualization; Software; Validation; Investigation; Data Curation; Writing – Original Draft; Visualization.

**Dr. Prabhakaran Abraham:** Review & Editing; Supervision; Project Administration.

## Ethics statements

Not applicable. This research did not involve human subjects, animal experiments, or data collected from social media platforms.

## Declaration of competing interest

The authors declare that they have no known competing financial interests or personal relationships that could have appeared to influence the work reported in this paper.

## Data Availability

No data was used for the research described in the article.
